# Interaction between etomidate and beta tumoral necrosis factor on hemodynamic response after cardiac surgery

**DOI:** 10.1186/cc14576

**Published:** 2015-03-16

**Authors:** JL Iribarren, JJ Jimenez, N Perez, M Brouard, R Perez, O Gonzalez, A Arbesu, R Martinez, ML Mora

**Affiliations:** 1 Hospital Universitario de Canarias, La Laguna, Spain

## Introduction

The use of etomidate is a risk factor for relative adrenal insufficiency in patients undergoing cardiopulmonary bypass (CPB) [1]. The objective was to determine the possible interaction between etomidate and beta tumoral necrosis factor (TNFβ) polymorphism on hemodynamics after CPB.

## Methods

A prospective cohort study on CPB patients who received etomidate or not during anesthetic induction during 2008 to 2011. Demographic and postoperative variables were collected. We tested the Hardy-Weinberg equilibrium in order to avoid selection bias. V18 SPSS was used.

## Results

**Figure 1:**
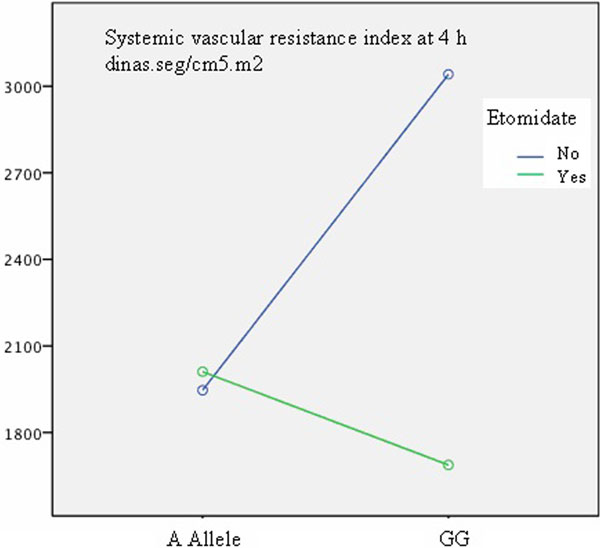


We studied 433 patients undergoing CPB, 285 (65.8%) men and 148 (34.2%) women, 66 ± 6 years, EuroSCORE I 5.3 ± 4%. TNFβ was in Hardy-Weinberg equilibrium (χ^2^: 0.6; *P *= 0.42). A total of 254 (58.7%) patients received etomidate, 152 out of them required vasoactive drugs. Homozygous G was defined as unfavorable TNFβ versus the A allele [2]. Using the general linear model after adjusting for sex and amines dose at 4 hours, an independent association was observed between the systemic vascular resistance index (SVRI) at 4 hours and the use of etomidate (*F*: 18; *P *< 0.001): 1,849 (95% CI: 1,673 to 2,024) versus 2,493 (95% CI: 2,258 to 2,729) dinas.seg/cm^5^.m^2^, the presence of homozygous G (*F*: 6.5; *P *= 0.01), and also showed a significant etomidate-homozygous G interaction (*F*: 22.8: *P *< 0.001): 1,687 (95% CI: 1,350 to 2,023) versus 3,041 (95% CI: 2,589 to 3,492) dinas seg/cm^5^. m^2^ (Figure 1).

## Conclusion

Etomidate use is associated with lower postoperative SVRI which is increased in the presence of G homozygosity for TNFβ polymorphism.
